# Aclacinomycin enhances the killing effect of allogeneic NK cells on acute myeloid leukemia cells by inducing immunogenic cell death

**DOI:** 10.3389/fimmu.2025.1521939

**Published:** 2025-02-20

**Authors:** Yongbin Ye, Ning Liu, Yunxin Zeng, Ziwen Guo, Xiaobo Wang, Xiaojun Xu

**Affiliations:** ^1^ Department of Hematology, Zhongshan Hospital Affiliated to Sun Yat-Sen University, Zhongshan, Guangdong, China; ^2^ Department of Hematology, The 6th Affiliated Hospital of Shenzhen University Health Science Center, Shenzhen, Guangdong, China; ^3^ Department of Hematology, The Seventh Affiliated Hospital, Sun Yat-Sen University, Shenzhen, Guangdong, China; ^4^ Internal Medicine Department, Tianyang People’s Hospital of Baise City, Baise, China

**Keywords:** aclacinomycin, allogeneic NK cells, acute myeloid leukemia, immunogenic cell death, HMGB1

## Abstract

**Introduction:**

Natural killer (NK) cells, which exert spontaneous cytotoxicity against infectious diseases and cancer, also play an important role in leukemia therapy. Despite the success of NK-based therapy in the treatment of myeloid leukemia, the potential use of NK alloreactivity in these hematologic malignancies remains elusive. The aim of the present study was to investigate whether allogeneic NK cells combined with aclacinomycin (ACM) could enhance anti-leukemic functionality against an acute myeloid leukemia (AML) cell line and to clarify the underlying mechanism.

**Methods:**

KG-1α and HL-60 AML cell lines were subjected to different treatments. The effects of different drug combinations on cytotoxicity, cell viability, and apoptotic status were examined.

**Results:**

The results showed that the combination of ACM (40 nmol/l) and allogeneic NK cells (ratio 20:1) was significantly cytotoxic to AML cells and increased the apoptosis of AML cells, especially after 72 h of treatment. Subsequent analyses revealed that the expression of immunogenic cell death (ICD)-related molecules calreticulin, adenosine triphosphate, and high mobility group box 1, as well as NK cell effector production—perforin and granzyme B—was markedly increased in the combination treatment group. These findings suggest that ACM enhances the anti-leukemic activity of allogeneic NK cells through the ICD pathway.

**Discussion:**

These results demonstrated that allogeneic NK cells had enhanced functional responses when stimulated with ACM *in vitro*, exhibiting superior effector cytokine production and cytotoxicity compared to the control, which contained conventional NK cells. In conclusion, the present study suggested that the combination of ACM and allogeneic NK cells is a promising therapeutic strategy against AML.

## Introduction

Acute myeloid leukemia (AML) is a hematological malignancy that can be classified by cytogenesis, molecular heterogeneity and an immunophenotype (1, 2). There are several main subtypes of AML, including AML with recurrent genetic abnormalities, AML with myelodysplasia-related changes, therapy-related myeloid neoplasms, AML-NOS (not otherwise specified), myeloid sarcoma and myeloid proliferations related to Down syndrome (2). Clinically, resistance to chemotherapy and relapse are still the main causes of poor outcomes in patients with AML. Anthracycline-based chemotherapy remains the standard first-line choice in adjuvant and palliative therapy, but the effect remains unsatisfactory (3). The failure of traditional therapeutic regimens for AML highlights a need for novel treatment strategies. Recently, immunochemotherapy has become a new hot spot in the treatment of cancer (4). It has been reported that certain chemotherapeutic drugs, such as anthracycline antineoplastic drugs (doxorubicin, epirubicin and idarubicin), may contribute to antitumor responses of immune cells by inducing a special form of tumor-cell killing, known as immunogenic cell death (ICD) (4–6). ICD-inducing drugs enhance tumor antigen exposure and promote the release of immunostimulatory tumor cell content-related proteins, such as calreticulin (CRT), adenosine triphosphate (ATP), and high mobility group box 1 (HMGB1) (7, 8). Therefore, the use of ICD-inducing agents can provide a convenient strategy for AML intervention (9, 10). In terms of using the ICD strategy for AML therapy, dendritic cells (DCs) and cytotoxic T cells are commonly studied immune effector cells, while the efficacy of natural killer (NK) cells has rarely been explored.

NK cells are important effector lymphocytes involved in anti-tumor response *in vivo* and play a central role in the immune surveillance of tumor and/or virally infected cells (11). NK cells exert a cytolytic effect on pathogen-infected or cancer cells by releasing cytolytic granule contents, notably perforin and granzyme B (12, 13). In addition, NK cells have been reported to activate other immune cells by secreting immunoregulatory cytokines, including interferon (IFN)-α (14), tumor necrosis factor-α, macrophage inflammatory protein-1, and regulated upon activation, normal T cell expressed and secreted (15). The activation of NK-cell effector functions is finely regulated by the recognition of stress-induced ligands or the mass activation of adhesion molecules or inhibitory receptors. In addition, NK cells have been used for adoptive cell therapy (16, 17). The clinical success of allogeneic hematopoietic stem cell transplantation (18), haploidentical transplantation (19), NK cell adoptive (20) transplantation, and monoclonal antibody therapy (21) have proven that NK cells play a key role in the treatment of AML. Although NK cells exhibited safety and efficacy in the treatment of patients with different malignancies (22, 23), the reactions were mostly transient and benefited only a small number of patients. The combination of allogeneic NK cells with chemotherapeutic agents is an attractive approach to improve and maximize tumor targeting and NK cell responsiveness (24).

Aclacinomycin (ACM) is an anthracycline anti-tumor antibiotic that is widely used in the treatment of solid tumors and hematological malignancies, such as lung (25) and breast (26) cancer, and AML (27). ACM exerts its anti-tumor activity through three different mechanisms: i) inhibiting the activity of topoisomerase II and leading to breaks in genomic DNA (28); ii) direct binding to tumor DNA and induction of DNA unwinding (29); and iii) inhibiting ubiquitin-ATP-dependent proteolysis in tumor cells (30). Furthermore, ACM acts as an “adjuvant” to enhance the efficacy of other anti-tumor drugs. Our previous study demonstrated that ACM combined with As_2_O_3_ displayed a synergistic killing effect on AML cells (27). However, the impact of ACM on NK-cell functionalities has not yet been well established. Andrade Mena et al. (31) reported that low-dose ACM enhances the activity of NK cells, while Santoni et al. (32) found that ACM could inhibit or stimulate NK cells depending on its dose and site of administration. Therefore, it remains unclear whether ACM treatment can enhance NK-cell mediated anti-tumor activity in patients with AML.

The aim of the present study was to investigate the treatment efficacy of the combination of ACM with allogeneic NK cell on AML cells. Through *in vitro* studies, the effects of ACM on allogeneic NK cell viability and functionalities, and the molecular mechanisms through which ACM enhances allogeneic NK cell killing activity toward AML cells were evaluated. Most importantly, it was demonstrated that ACM can enhance the killing activity of allogeneic NK cells against AML cells through “ICD” signaling. Furthermore, this study revealed that allogeneic NK cells and ACM can potently work together against AML cells, providing a possibility to explore this combination strategy in AML treatment.

## Materials and methods

### Cell culture

The KG-1α acute myeloid leukemia cell line [CCL-246.1™, American Type Culture Collection, (ATCC)] was kindly provided by Professor Zengxuan Song (Chinese Academy of Medical Sciences and Peking Union of Medical College, China). An important phenotypic abnormality of KG-1α cells is their early or primitive developmental stage and inability to differentiate into functionally mature cells (33). Due to these characteristics, KG-1a cells were often used in AML research. The HL-60 acute myelogenous leukemia cell line was purchased from the ATCC (CCL-240™). HL-60 cells, derived from human promyelocytic leukemia, share some similarities with promyelocytic cells and can differentiate into different cell types (34). The cell culture method was based on previously reported procedures (31).

### CD56^+^CD3^-^ NK cell generation

NK cells were isolated from peripheral blood mononuclear cells (PBMNCs) obtained from unidentified, healthy donors. All donors provided written informed consent. The study was approved by the Ethics Committee of Zhongshan People’s Hospital Affiliated to Sun Yat-sen University (Approval No.: K2017-003) and was conducted in accordance with the Declaration of Helsinki. The culture protocol was adapted from Cany et al. (35). CD56^+^CD3^-^ NK cells were magnetically isolated using human CD56 (Catalog number: 130-050-401) and CD3 (Catalog number: 130-050-101) MicroBeads (Miltenyi Biotec GmbH). In brief, human peripheral blood was collected from healthy volunteers using heparin as an anticoagulant. After a doubling dilution with PBS, density gradient centrifugation (800 g for 30 minutes in a swing-out rotor at 18-22°C) with human lymphocyte separation solution (Catalog number: 20828-15, Nacalai Tesque, Inc.) was conducted, and mononuclear cells were collected from the interface and washed. Cells were then placed in a plastic pipe (5x10^6^/ml) and treated with pectin methylesterase (PME; 5 mmol/l; Catalog number: 9025-98-3, Sigma-Aldrich) at 37°C for 40 min to remove B cells and monocytes/macrophages. The remaining PME-treated PBMNCs (5x10^6^ cells/ml) were cultured in RPMI-1640 (Catalog number: 11875093, Thermo Fisher Scientific, Inc.) with 22 nM (6,000 IU/ml) interleukin (IL)-2 (MQ1-17H12; BD Biosciences) for 4-5 h. CD56^+^ NK cells adhering to the surface of the plastic culture flask were collected and re-cultured for 4 weeks. CD56+ cells purity was quantified by adding 1x10^5^ cells into the flow cytometry (FCM, CytoFLEX, Beckman Coulter, Brea) and the flow cytometric data were evaluated by the FlowJo V10.6.3 software (BD Life Sciences–FlowJo). The cell viability was determined using a colorimetric WST-8 dehydrogenase assay [cell counting kit-8 (CCK-8); catalog number: CK04-11, Dojindo Molecular Technologies, Inc.]. The use of allogeneic NK cells at the end of the culture process typically results in CD56^+^ purity exceeding 80% within 4 weeks of culture.

### Cell viability assay following ACM treatment

ACM was purchased from Enzo Life Sciences, Inc. For *in vitro* studies, ACM was dissolved in dimethyl sulfoxide (Catalog number: 276855-1L, Sigma-Aldrich) at 10 mmol/l and stocked at -20°C. The inhibition of KG-1α and HL-60 cell viability by ACM was assessed using the CCK-8 assay, according to the manufacturer’s instructions. The detection of cell viability was carried out as previously described (32).

### Cell viability assay following combination treatment with ACM and NK cells

KG-1α and HL-60 cells collected at the logarithmic growth phase were washed three times with RPMI-1640 medium supplemented with 10% FBS (Catalog number: F2442-500ML, Sigma-Aldrich) before being used as target cells, and cell density was adjusted to 2x10^5^/well in 6-well plates. NK cells (effector cells) were added to the 6-well plates (4x10^6^/well) and induced by ACM at a final concentration of 40 nmol/l. Mixed culture cells without ACM treatment were used as the control. Following incubation for 24, 48, 72 or 96 h, the NK cell-mediated killing effect of the susceptible KG-1α and HL-60 cell line was measured using lactate dehydrogenase (LDH) release (LDH cytotoxicity assay kit, catalog number: 11644793001, Roche Diagnostics) (36). The LDH levels were determined using an automatic biochemical analyzer (Model 170A; Hitachi, Ltd.), and the killing activity of NK cells in each group was calculated according to the formula presented in the manual of the LDH detection kit. The percentage of cytotoxicity was calculated using the following formula: [(Experimental release - spontaneous release)/(total release - spontaneous release)] x 100% (37).

### AML cell apoptosis

Based on the results of the CCK-8 and LDH assays, the treatments with 40 nmol/l ACM and NK cells at an effector to target ratio of 20 were selected, in order to analyze their effect on AML cell and PBMNCs apoptosis. KG-1α, HL-60 cells and PBMNCs were added to 6-well plates (2x10^5^ cells/well) and were untreated (control group) or treated with ACM combined with NK cells, ACM alone or NK cells alone (5 wells/group). KG-1α and HL-60 cells were collected following incubation for 24, 48, 72 and 96 h. Cells were then harvested and washed twice with ice-cold PBS and resuspended in 1x Binding Buffer at a concentration of 6 x 10^5^ cells/ml. A total of 200 µl of the mixtures were then transferred to a 1.5 ml culture tube and stored on ice, then 5 ml of Annexin V-FITC (Catalog number: APOAF-60TST, Sigma-Aldrich) and 5 ml of propidium iodide (PI, catalog number: P1304MP; Thermo Fisher Scientific, Inc.) were added to the culture tube. The cells were gently vortexed and incubated for 10 min at room temperature in the dark, and stained cells (1.2 x 10^5^) were analyzed by FCM (CytoFLEX, Beckman Coulter, Brea) to determine the percentages of Annexin V+/PI- (early apoptotic) and Annexin V+/PI+ (late apoptotic) cells by the FlowJo V10.6.3 software (BD Life Sciences–FlowJo) (38).

### Western blotting

Anti-CRT (Catalog number: MBS2006516), anti-heat shock proteins (Catalog number: VPA00800), anti-ATP (Catalog number: MBS2041256) and anti-HMGB1 (Catalog number: GTX101277) antibodies were purchased from Biocompare, Inc. Anti-perforin (Catalog number: MA5-12469), anti-granzyme B (Catalog number: GRB04), anti-β-actin (Catalog number: MA1-140) antibodies, and goat anti-rabbit horseradish peroxidase-conjugated secondary antibodies (Catalog number: 31460) were purchased from Thermo Fisher Scientific, Inc. For western blotting, treated and untreated cells were homogenized in 150 µl ice-cold lysis buffer on ice for 30 min. Subsequently, the homogenates were boiled and subsequently centrifuged at 300 g at 20°C. The supernatant-containing proteins were then analyzed by immunoblotting. Markers of ICD, such as CRT, ATP and HMGB1 were analyzed. In addition, perforin and granzyme B-the two direct evidences of the killing activity of activated NK cells, were also analyzed in the HL-60 cell culture. Quantification analysis of the western blot results were performed via ImageJ (National Institutes of Health).

### Statistical analysis

Data on cell viability, apoptotic ratio and quantification of western blotting are presented as the mean ± SD. One-way analysis of variance followed by Tukey’s test for multiple comparisons was performed to assess the differences between two groups. GraphPad Prism (version 8.0.2, GraphPad Software, Inc.) was used for data processing. P<0.05 was considered to indicate a statistically significant difference. All the experiments were independently repeated 3 times.

## Results

### Purity of sorted human NK cells

**Figure 1:**
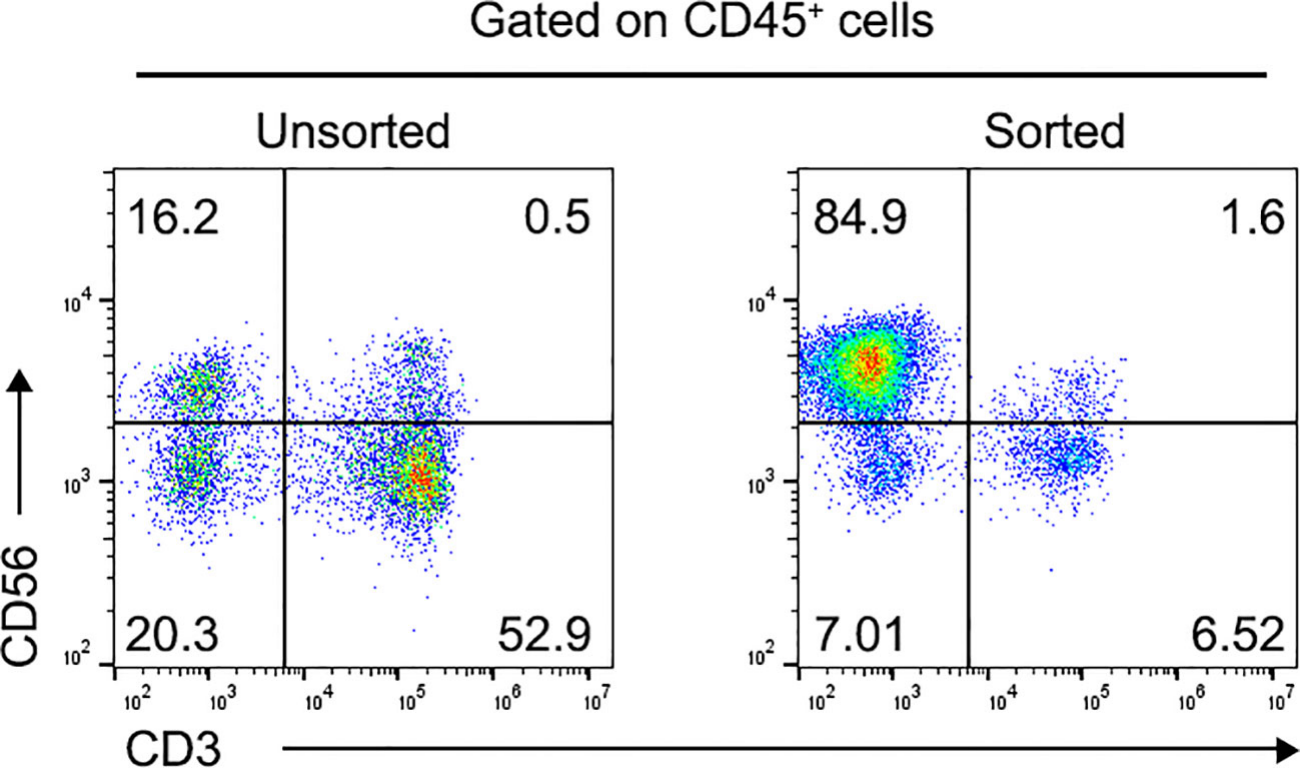
Proportion of CD3^-^CD56^+^ NK cells in human PBMNCs. Flow cytometric analysis of CD3^-^CD56^+^ NK cells using annexin V/FITC staining before (Left) and after (Right) CD56^+^ cell positive selection. Data was presented as the mean ± SD of three independent experiments. NK, natural killer; PBMNCs, peripheral blood mononuclear cells.

The percentage of CD3^-^CD56^+^ NK cells in the collected PBMNCs was 15.2 ± 3.7% before sorting (**Figure 1**). Following a 4-week culture period, this proportion significantly increased, with CD3^−^CD56^+^ NK cells constituting 84.9 ± 5.5% of the total cell population (**Figure 1**).

### Cytotoxic effect of ACM on AML cell lines *in vitro*

**Figure 2:**
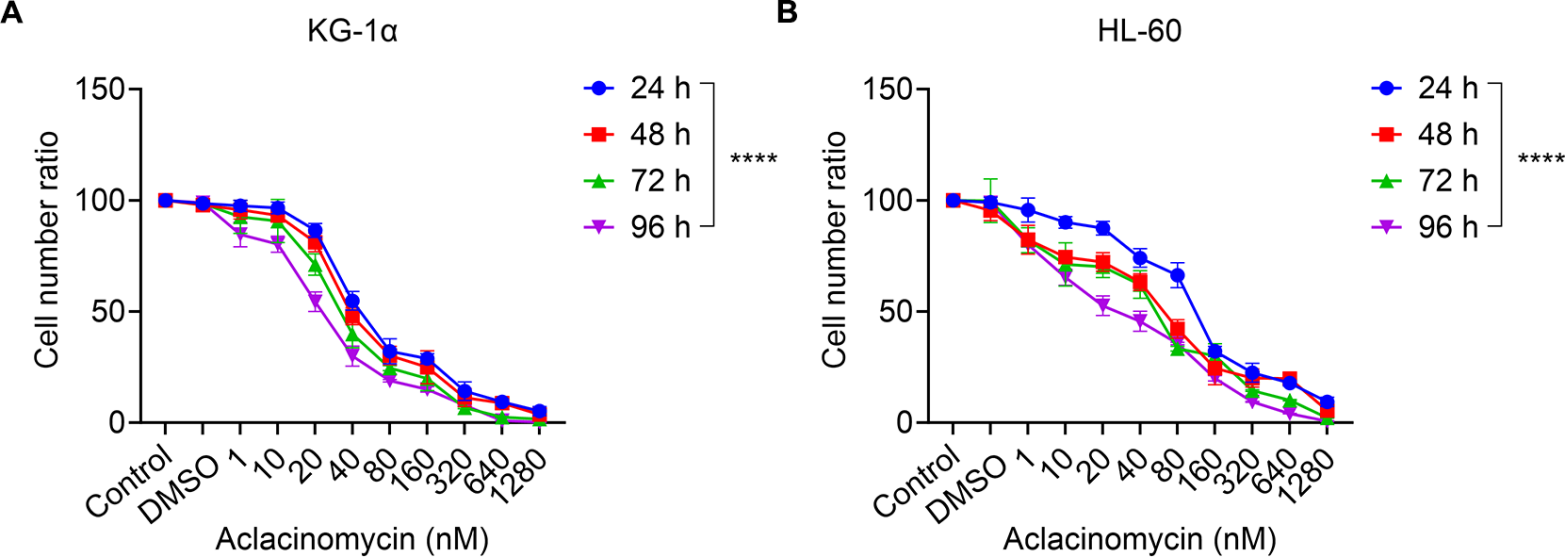
Effects of ACM treatment on the metabolism of KG-1α and HL-60 cells *in vitro*. **(A)** KG-1α and **(B)** HL-60 cells were treated with different concentrations of ACM (1-1,280 nmol/l) for 24, 48, 72 and 96 h, and cell viability was evaluated using the CCK-8 assay. AML cells were pre-incubated with 1-1,280 nmol/l ACM, blank control (Control: without ACM treatment) or negative control (DMSO). Data was presented as the mean ± SD of three independent experiments. ****P < 0.0001. ACM, aclacinomycin; CCK-8, cell counting kit-8; AML, acute myeloid leukemia; DMSO, dimethyl sulfoxide.

To explore the potential of combining allogeneic NK cells with ACM therapy, the cytotoxic effects of ACM on two AML cell lines were initially assessed. KG-1α and HL-60 cells were treated with ACM at concentrations ranging from 0 to 1,280 nM, and cytotoxicity was evaluated using the CCK-8 assay. As anticipated, ACM treatment resulted in a time- and dose-dependent reduction in KG-1α cell viability compared to untreated controls, with significant decreases observed over 24 to 96 hours at varying concentrations (**Figure 2A**). Similar trends were noted in HL-60 cells (**Figure 2B**). These findings indicate that ACM concentrations between 0 and 1,280 nM moderately inhibited the viability and proliferation of KG-1α and HL-60 cells. However, when the ACM concentration exceeded 640 nM, minimal changes in cell viability and proliferation were observed. Therefore, to minimize the effects of excessive cytotoxicity, ACM concentrations of 0-640 nM were selected for subsequent experiments.

### ACM enhances the killing effect of allogeneic NK against AML cells *in vitro*

**Figure 3:**
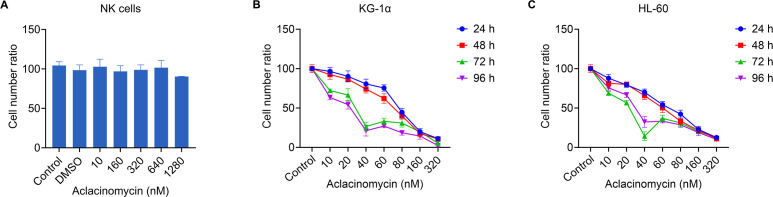
Effects of combination treatment (allogeneic NK cells and ACM) on the metabolism of KG-1α and HL-60 cells. **(A)** The effects of different concentrations of ACM on NK cell viability after 96 hours of treatment were assessed using the CCK-8 assay. The control group consisted of cells without ACM treatment, and DMSO was used as a negative control. **(B)** KG-1α and **(C)** HL-60 cells were treated with ACM (0-320 nmol/l) and NK cells (effector to target ratio was 20) for 24, 48, 72 and 96 h, and cell viability was analyzed using an LDH assay. AML cells were pre-incubated with 10-320 nmol/l of ACM or Control (without ACM treatment). Data are presented as the mean ± SD of three independent experiments. NK, natural killer; ACM, aclacinomycin; LDH, lactate dehydrogenase; AML, acute myeloid leukemia.

Next it was investigated whether the treatment of AML cells with ACM affected their susceptibility to allogeneic NK cell-mediated cytotoxicity. To exclude the potential impact of ACM on the viability of effector cells (NK cells), we evaluated the effects of different concentrations of ACM on NK cells after 96 hours of treatment using the CCK-8 assay. The results showed that ACM exhibited some growth-inhibitory effects on NK cells at a concentration of 1290 nM, although the difference was not statistically significant (**Figure 3A**). In subsequent co-culture cytotoxicity experiments, the ACM concentration was set at 320 nM. Therefore, the influence of ACM on the viability of effector cells (NK cells) can be ruled out. Therefore, KG-1α and HL-60 cells were treated with a combination of ACM (0-640 nmol/l) and used as targets for allogeneic NK cells (effector to target ratio was 20), and cell viability was determined at different timepoints using the CCK-8 assay. At the start of the co-culture, equal numbers of KG-1α and HL-60 cells were seeded under each experimental condition. KG-1α and HL-60 cell survival assays suggested that the effects of ACM and allogeneic NK cells were at least additive. Following co-culture with ACM and allogeneic NK cells, KG-1α cell viability was decreased in a time- and dose-dependent manner with increasing ACM drug concentrations (**Figure 3B**). Furthermore, similar results were obtained with the HL60 cell line (**Figure 3C**). Of note, the survival of KG-1α and HL-60 cells was most significantly decreased when these cells were treated with allogeneic NK cells and 40 nM of ACM for 72 h. Thus, this concentration was selected for subsequent experiments. These data supported that ACM could enhance the AML cell-mediated killing of allogeneic NK cells, particularly in a specific range of concentrations, and that the effects of allogeneic NK cells and ACM are complementary, effectively reducing the number of AML cells when the two are combined.

### The effect of ACM and allogeneic NK cells on AML cells apoptosis

**Figure 4:**
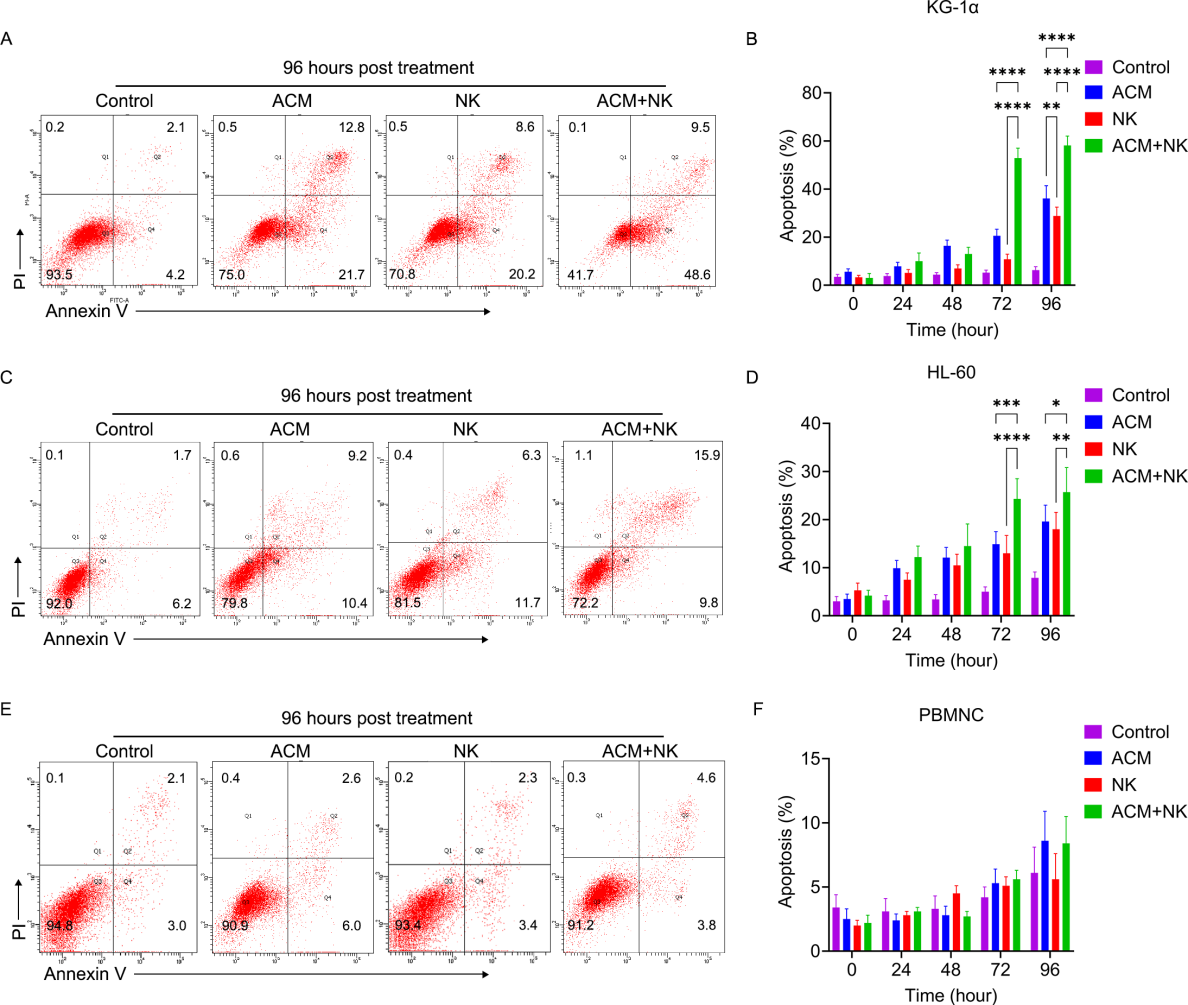
Representative flow cytometry images of **(A)** KG-1α cells, **(C)** HL-60 and **(E)** PBMNC following combination treatment with allogeneic NK cells and ACM. ACM (40 nmol/l) and allogeneic NK cells (effector to target ratio was 20) were administered alone or together to KG-1α cells and the incubation time ranged from 24 to 96 h. Afterwards, the apoptosis of these cells was determined by FCM. Quantitative analysis of the apoptotic ratio of the **(B)** KG-1α, **(D)** HL-60 and **(F)** PBMNC after the combination treatment of allogeneic NK cells and ACM. Data are presented as the mean ± SD. ^*^P<0.05, ^**^P<0.01, ^***^P<0.001 and ^****^P<0.0001.

To further determine how the potentiating effect of ACM on NK cells occurs, the apoptosis of KG-1α and HL-60 cells, and normal PBMNCs following treatment with ACM, allogeneic NK cells or a combination of both (**Figures 4–E**). To do this, 40 nmol/l ACM together with allogeneic NK cells at an effector to target ratio of 20 was used to treat KG-1α and HL-60 cells, and normal PBMNCs based on the results of the previous step, as this combination can yielded marked inhibitory effects.

First, the apoptotic ratio of KG-1α cells following ACM treatment was examined using FCM (**Figures 4A, B**), and it was found to be increased as the treatment time increased from 24 to 72 h. Following allogeneic NK cell treatment (**Figures 4A, B**), the apoptotic ratio was slightly increased and only the ratios at the 72- and 96-h timepoints were found to be higher than the ratio at the 24-h timepoint. The combination treatment of ACM and allogeneic NK cells induced a marked improvement in the apoptotic ratio of the cells at the 72-h timepoint, which was maintained until the 96-h timepoint (**Figure 4B**). The combination treatment group exhibited the highest cell apoptosis among the three groups at both the 72- and 96-h timepoints.

The apoptotic ratio of the HL-60 cells following ACM treatment (**Figures 4C, D**) was significantly increased from 24 to 96 h. The allogeneic NK cell treatment also induced a slight improvement in the apoptotic ratio. The combination treatment groups induced a marked increase in apoptosis from the 24- to the 72-h timepoint. There were no significant differences between the values at 72 and 96 h from treatment. Following a comparison of the three treatment groups, the combination group showed the highest apoptotic ratios from the 48- to the 96-h timepoint. In combination, these results demonstrated that ACM can potentiate allogeneic NK cell cytotoxicity, suggesting that the combination of allogeneic NK cells with ACM could lead to synergistic effects against AML cells. Of note, the combination of allogeneic NK cells with ACM did not trigger a significant increase in the apoptotic ratios of normal PBMNCs following treatment for 24-96-h (**Figures 4E, F**).

### Upregulation of ICD-related molecules in AML cells by combination treatment of ACM and allogeneic NK cells

**Figure 5:**
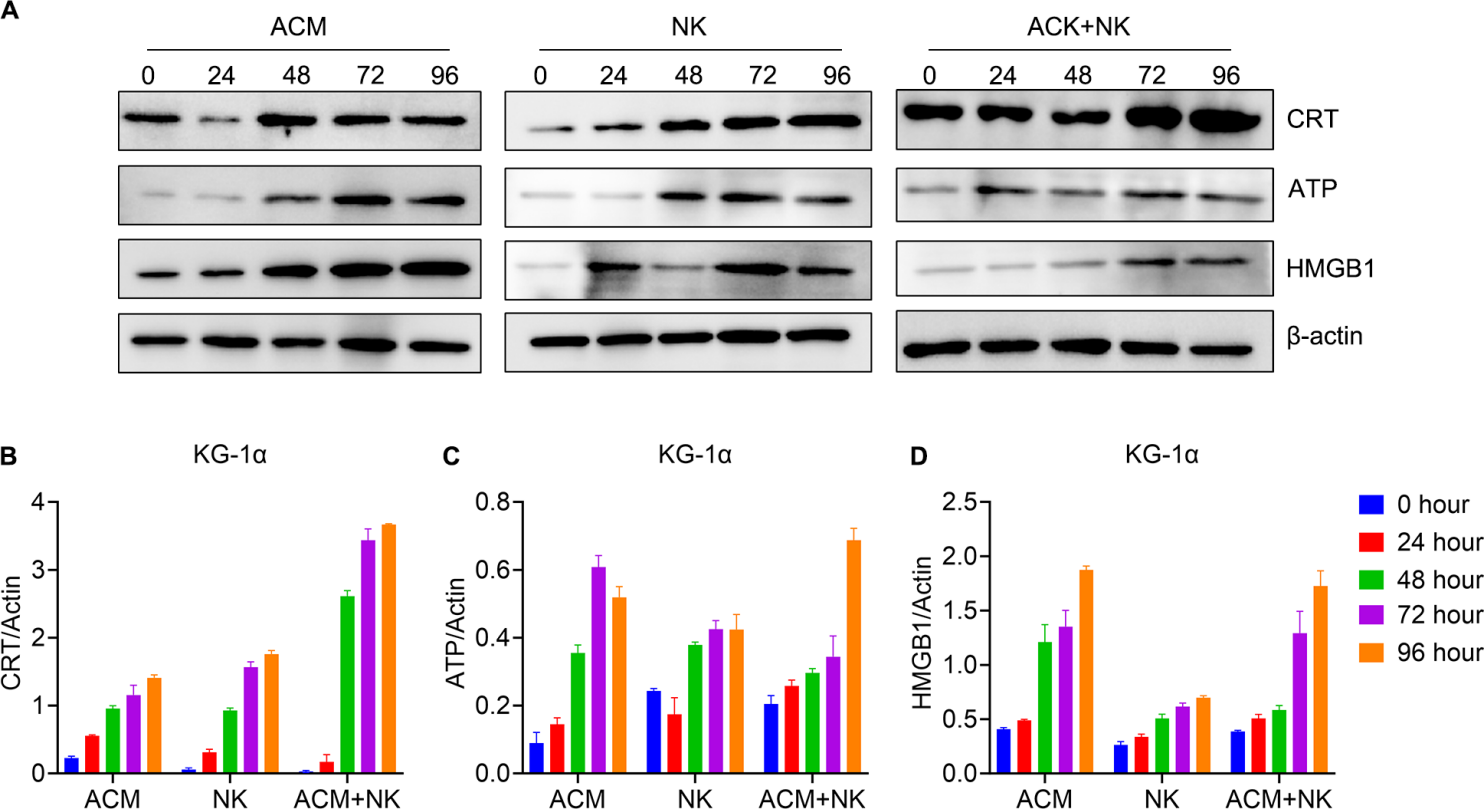
Modulation of the expression of ICD pathway-related molecules in KG-1α cells following combination treatment with ACM and allogeneic NK cells. **(A)** Western blotting of CRT, ATP and HMGB1. **(B-D)** Quantification analysis of the western blotting results for CRT, ATP and HMGB1 respectively. ACM (40 nmol/l) and allogeneic NK cells (effector to target ratio was 20) were administered alone or together to the KG-1α cells and the incubation time ranged from 24 to 96 h. The levels of CRT, ATP and HMGB1 were then analyzed by western blotting, followed by ImageJ quantification. CRT, calreticulin; ATP, adenosine triphosphate; HMGB1, high mobility group box 1.

Earlier studies have reported that anthracycline antineoplastic drugs may contribute to antitumor responses by inducing a special form of tumor-cell killing, known as ICD. We asked whether the combination treatment with ACM and allogeneic NK cells induce ICD to a similar extent. Therefore, the levels of ICD-related molecules in AML cells were analyzed by western blotting following combination treatment with ACM and allogeneic NK cells (**Figure 5A**). Regarding the protein expression in the KG-1α cells (**Figures 5B–D**), the results showed that the combination group displayed a much higher CRT level compared to that in the ACM and NK groups at the 48-, 72- and 96-h timepoints. The combination group exhibited a higher ATP expression than that in the other two groups at the 96-h timepoint. No statistical differences in HMGB1 expression were observed between the combination and ACM group at the 72- and 96-h timepoints.

**Figure 6:**
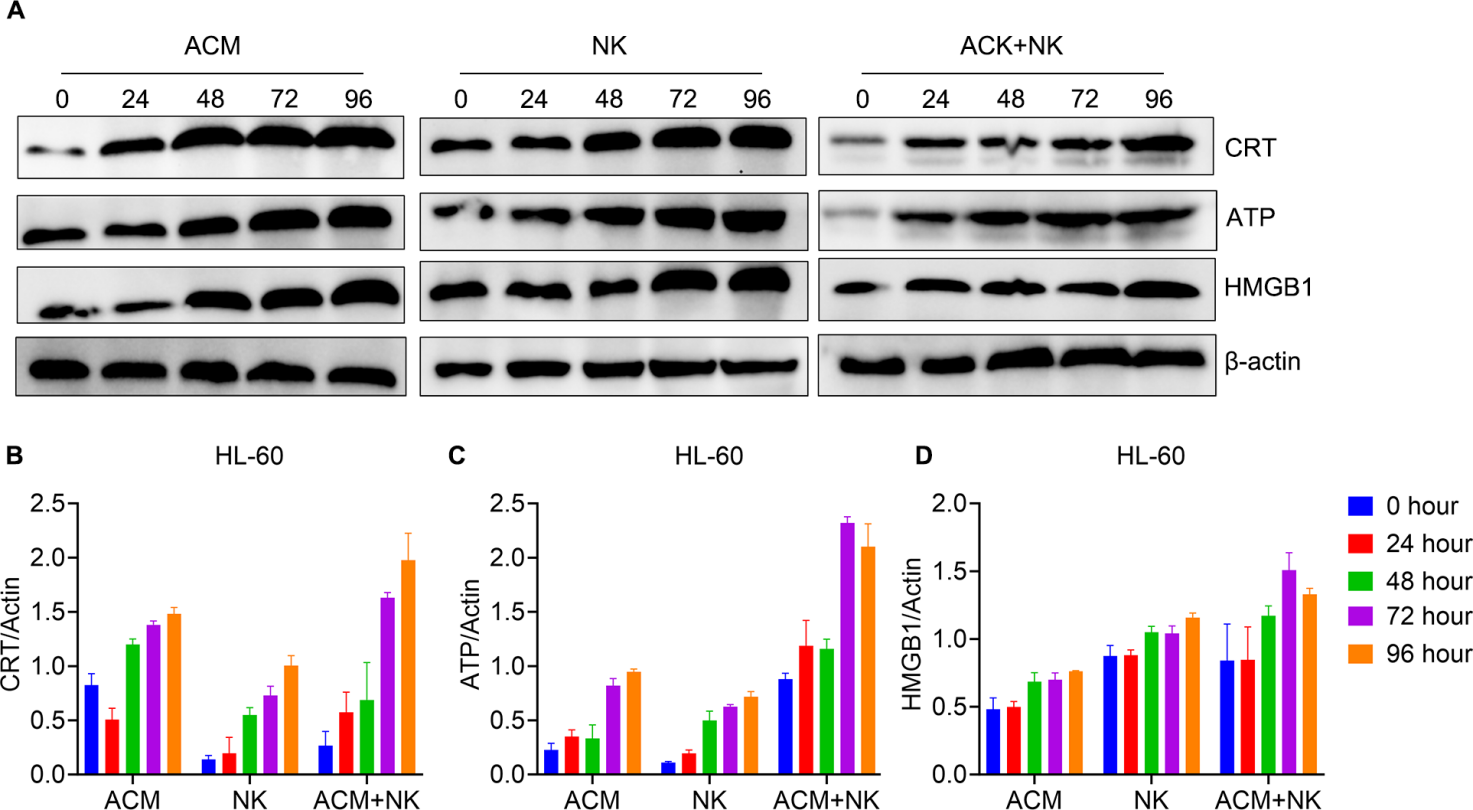
Modulation of ICD pathway related molecules expression of HL-60 cells by the combination treatment of ACM and allogeneic NK cells. **(A)** Western blotting of CRT, ATP and HMGB1. **(B-D)** Quantification analysis of CRT, ATP and HMGB1 from western blotting results, respectively. ACM (40 nmol/l) and allogeneic NK cells (effector to target ratio was 20) were administered alone or together to the KG-1α cells and the incubation time ranged from 24 to 96 h. The levels of CRT, ATP and HMGB1 were analyzed by western blotting, followed by ImageJ quantification.

**Figure 6** showed the protein expression of the HL-60 cells after various treatments. The CRT levels of the 72- and 96-h timepoints of the combination treatment group were significantly higher than the ones of other groups at the same timepoints (**Figures 6A, B**). In turns of the ATP expression, the combination group showed the highest ATP expression at the 48- to 96-h timepoints among the groups of the same timepoints (**Figure 6C**). For the HMGB1 expression, the values of the 48- and 96-h timepoints of the combination group were obvious higher than the ones of the other two groups at the same timepoints (**Figure 6D**). Additionally, the value of the 72-h was the highest among the three groups for the same timepoint. These data confirm that ACM enhances the killing effect of allogeneic NK cells on AML cells via inducing ICD.

### Activation of allogeneic NK cells by ACM

**Figure 7:**
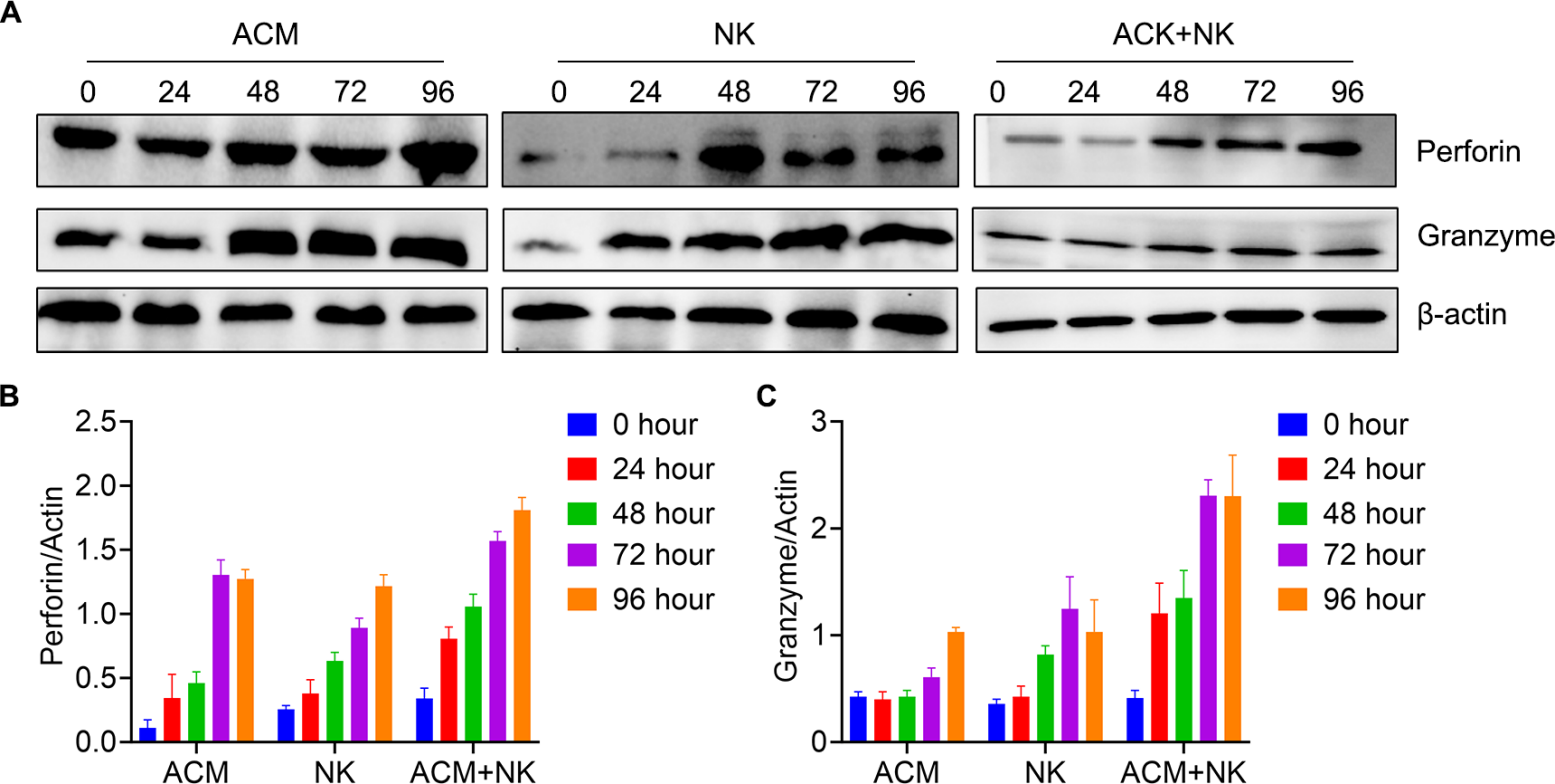
Activation of allogeneic NK cells by ACM. **(A)** Western blotting for perforin and granzyme B. **(B)** Quantification of perforin based on the western blotting results. ACM (40 nmol/l) and allogeneic NK cells (effector to target ratio was 20) were administered alone or together to HL-60 cells, with incubation times ranging from 24 to 96 h. **(C)** Quantification of granzyme B based on the western blotting results. The same conditions as in **(B)** were applied for granzyme B. The levels of perforin were quantified using ImageJ.

The release of perforin and granzyme B is direct evidence of the killing effect of NK cells following activation (39). Therefore, the detection of the levels of perforin and granzyme B proteins can effectively demonstrate whether allogeneic NK cells are activated. Thus, the levels of perforin and granzyme B of HL-60 cells following treatment were tested. In **Figure 7**, it is shown that the perforin expression was increased from 24 to 72 h and maintained after 96 h through combination treatment (**Figures 7A, B**). The combination treatment group showed the highest value among the three groups at the same timepoints. Granzyme B expression was higher in the combination group compared with the other two treatment groups at the 24, 48 and 72 h (**Figures 7A, C**).

## Discussion

ACM has been widely used in the treatment of hematological malignancies and solid tumors; however, its mechanism in cancer treatment is not entirely clear. It has been reported that ACM can either increase or decrease NK-cell activity depending on the administered dose (32). Herein, the capacity of ACM to enhance the allogeneic NK cell killing activity against AML cells was investigated. The present results clearly indicated that the combination of ACM and NK cells can synergistically inhibit the viability and induce the apoptosis of AML cells. Furthermore, these results proved that the superior killing effect of the combination treatment on AML cells was due to the induction of ICD and the activation of NK cells via ACM. Therefore, the present study may provide new prospects for the treatment of AML.

Several studies have shown that NK cell-mediated AML therapy following hematopoietic stem cell transplantation is safe (40–42). However, only a limited therapeutic effect was showed when NK cells were used as monotherapy (43), which may be associated with the different immune escape mechanisms of AML cells which may lead to the dysfunction of NK cells (44, 45). Furthermore, the differentiation of NK cells may be inhibited by tumor cells (41, 46). Therefore, it is critical to reactivate NK cells during the treatment of AML (47). It has been proven that the reactivation of NK cells may be induced by certain drugs, including cytotoxic drugs, immune adjuvants and IFN. Previous studies have shown that ACM combined with other drugs can enhance the toxicity to tumor cells (27, 31). However, little is known about the combination of ACM and allogeneic NK cells against AML. Hence, the effect of combination treatment with allogeneic NK cells and ACM on the viability and proliferation of KG-1α and HL-60 cells. These results showed that, as compared with NK cell treatment alone, the combination treatment induced marked viability inhibition that was time- and dose-dependent. Of note, KG-1α and HL-60 cell metabolism was inhibited to a greater extent when ACM (40 nmol/l) was combined with NK cells at an effector to target ratio of 20. The same ACM concentration also contributed the most to the killing effect of allogeneic NK cells on AML cells, which was consistent with previously reported results (31).

In the next experiment, the possible mechanism underlying the synergistic killing effect of allogeneic NK cells and ACM on AML cells was explored. A previous study has shown that certain chemotherapeutic drugs can provoke ICD and lead to cancer cell apoptosis (48). Through this pathway, chemotherapeutic drug-induced tumor cell autophagy emits three signals: CRT exposure on the cell surface to stimulate DC phagocytosis, ATP release to recruit DCs and HMGB1 release to promote the stable binding of DCs to dying tumor cells and induce anti-tumor immune responses (49). The ICD-related markers were evaluated in this study. It was found that the levels of CRT, ATP and HMGB1 of HL-60 cells following the combination treatment were significantly higher than those in the control and monotherapy groups. It is still unclear why the combination treatment only induced higher CRT and ATP levels, but not the HMGB1 level in the KG-1α cells compared with single treatments. Currently, the combination of different concentrations of ACM and the ratios of effector/target cells on the KG-1α cells was explored. Nevertheless, these results suggested that ACM can activate the ICD pathway in AML cells, which in turn can enhance the anti-AML activity of allogeneic NK cells.

Konjevic et al. (23) reported the various functions and modulation of NK cells in different malignancies. The role of NK cells in anti-tumor adoptive cell immunotherapy has attracted increasing interest in recent years (22). NK cells are not only non-specific immune cells that can kill tumor and virus-infected cells, but also effective in the treatment of AML and acute lymphoid leukemia with a poor prognosis (50–52). This effect of NK cells is mediated by toxic molecules, such as perforin and granzyme B, released following NK cell activation. When NK cells get in contact with their target, perforin is released to form a tubular channel of polyperforin, triggering the lysis and destruction of target cells (53). Granzyme B is the most important serine protease contained in the granules of NK cells. This protein can enter the target cells and activate a caspase cascade, causing DNA breakage and apoptosis (54). In the present study, the activation of allogeneic NK cells by ACM was confirmed in the HL-60 culture. The present data showed that the killing activity of NK cells on AML cells following activation of NK cells by ACM *in vitro*.

In conclusion, the present study comprises the first systematic and in-depth analysis of NK cell-mediated anti-leukemia responsiveness through the activation of ACM *in vitro*. It was also confirmed that this activation may follow the ICD pathway. Though the specific mechanism of ACM-enhanced killing activity of the NK cells needs to be further elucidated, this study provided a deeper understanding and an experimental basis for targeted therapy for AML through allogeneic NK cell-mediated anti-tumor immune activation.

## Data Availability

The raw data supporting the conclusions of this article will be made available by the authors, without undue reservation.
